# Assessment of the effectiveness of BG-Sentinel traps baited with CO_2_ and BG-Lure for the surveillance of vector mosquitoes in Miami-Dade County, Florida

**DOI:** 10.1371/journal.pone.0212688

**Published:** 2019-02-22

**Authors:** André B. B. Wilke, Augusto Carvajal, Johana Medina, Melissa Anderson, Veronica J. Nieves, Monica Ramirez, Chalmers Vasquez, William Petrie, Gabriel Cardenas, John C. Beier

**Affiliations:** 1 Department of Public Health Sciences, Miller School of Medicine, University of Miami, Miami, FL, United States of America; 2 Miami-Dade County Mosquito Control Division, Miami, FL, United States of America; Faculty of Science, Ain Shams University (ASU), EGYPT

## Abstract

Vector-borne diseases are an increasing issue to public health, endangering billions of people worldwide. Controlling vector mosquitoes is widely accepted as the most effective way to prevent vector-borne disease outbreaks. Mosquito surveillance is critical for the development of control strategies under the integrated vector management framework. We hypothesize that the effectiveness and reliability of using BG-Sentinel traps for the surveillance strongly depend on the bait used to attract mosquitoes. The objective of this study was to compare the effectiveness of BG-Sentinel traps baited with CO_2_ and BG-Lure. A total of 72 traps were deployed for 48 hours once a week for four weeks. For the initial 24-hour period, the traps were baited with CO_2_, and then for an additional 24 hours using the BG-Lure. Collected mosquitoes were analyzed using the Generalized Estimating Equation for repeated measures analysis. Biodiversity was assessed by the Shannon and Simpson indices and by individual rarefaction curves and SHE profiles. A total of 5,154 mosquitoes were collected, from which 3,514 by traps baited with CO_2_ and 1,640 mosquitoes by traps baited with BG-Lure. *Aedes aegypti* and *Culex quinquefasciatus* were the most abundant and dominant species. Results from the Generalized Estimating Equation models indicated that more than twice as many mosquitoes were attracted CO_2_ than to the BG-Lure. The comparison of attractiveness of CO_2_ and BG-Lure to *Ae*. *aegypti* and *Cx*. *quinquefasciatus* was non-significant, suggesting that both species were equally attracted by the baits. The individual rarefaction curves for *Ae*. *aegypti* and *Cx*. *quinquefasciatus* imply that traps baited with BG-Lure underestimated mosquito species richness compared to those baited with CO_2_. BG-Lure were less effective in attracting mosquitoes with low abundances and failed to collect *Cx*. *coronator* and *Cx*. *nigripalpus*, which were consistently collected by traps baited with CO_2_. According to our results, CO_2_ significantly (*P*<0.05) attracted more mosquitoes (2.67 adjusted odds ratios) than the BG-Lure when adjusted for time and species, being more effective in assessing the relative abundance of vector mosquitoes and yielding more trustworthy results. Traps baited with CO_2_ collected not only more specimens, but also more species in a more consistent pattern.

## Introduction

Vector-borne diseases (VBD) are an increasing problem to public health [1–3]. Billions of people worldwide are at risk of being infected by arboviruses, and millions of cases are reported every year. Recent estimates indicated that the dengue virus (DENV) endangers more than 3 billion people globally, infecting more than 390 million people every year [4]. The Zika virus (ZIKV) outbreak in 2016 took the world by storm, the estimated number of cases in the American continent revolves around 700,000 cases, distributed among the 45 countries that have reported local transmission of ZIKV [5–7]. Notwithstanding the morbidity caused by the infection, ZIKV was also responsible for pregnancy and congenital neurologic malformations in fetuses [8–10].

Controlling mosquitoes is accepted as the most effective way to prevent VBD outbreaks [11]. However, it is not an easy task. *Aedes* (*Stegomyia*) *aegypti* (Linnaeus), *Aedes* (*Stegomyia*) *albopictus* (Skuse), *Culex* (*Culex*) *quinquefasciatus* Say and *Culex* (*Culex*) *nigripalpus* Theobald are among the most adapted species to live alongside humans in urban environments. They are exceptionally adapted to thrive in urban and suburban areas, blood feeding in human hosts and laying eggs in artificial breeding sites, widely benefiting from anthropogenic alterations in the environment [12–16]. *Aedes aegypti* and *Ae*. *albopictus* are the primary vector for many arboviruses, including DENV, chikungunya virus (CHIKV), yellow fever virus (YFV) and ZIKV [5,17–21]. *Aedes albopictus* was also found infected with YFV in a transmission hotspot in Brazil. However, *Haemagogus leucocelaenus* is considered the main vector of YFV in Brazil and further investigation is still needed to define the role of *Ae*. *albopictus* in the transmission of YFV to humans [22,23]. *Culex quinquefasciatus* and *Cx*. *nigripalpus* are the primary vectors for, among others, West Nile virus (WNV) and Eastern Equine encephalitis (EEE) [24–27].

Vector-borne disease outbreaks are becoming more frequent in previously non-endemic areas, notably the ZIKV outbreak in the Americas [5], and the outbreak of CHIKV in Italy [28–30]. Due to the lack of vaccines and drugs for most arboviruses and the unfeasibility of stopping travelers carrying arbovirus from coming and going, controlling mosquito populations is the only feasible alternative.

The integrated vector management (IVM) is the gold standard for controlling mosquitoes [11]. It encompasses the use of scientifically-driven strategies to control mosquito populations, taken into account the ecosystem, management of breeding sites, education of the general public and the use of insecticides to control adult mosquito populations when needed. Many new strategies for controlling mosquitoes have been proposed, however, according to the Vector Control Advisory Group (VCAG) of the World Health Organization (WHO) none of them has yet been proven effective and safe to be included under the IVM framework [31,32]. Therefore, rendering mosquito surveillance and traditional control strategies an essential part of IVM.

Having a reliable surveillance network of traps to assess the relative abundance of vector mosquitoes in a given area is critical to inform control actions and prevent outbreaks. BG-Sentinel traps (BioGents, Regensburg, Germany) baited with BG-Lure (BioGents, GmbH, Regensburg, Germany) as attractant are considered the gold standard for collecting *Aedes* vectors, especially from the subgenus *Stegomyia* [33,34]. Previous studies have also reported the effectiveness of BG-Sentinel traps baited with BG-Lure in collecting *Culex* species [35,36], especially when baited with CO_2_ in addition to the BG-Lure [37–39].

Miami-Dade County, Florida is a major gateway to the United States with an increased number of people coming and going from and to endemic areas. Therefore, the development of a reliable and dependable surveillance system is critical to guide and support a successful surveillance program aimed to prevent future outbreaks, both in Miami and elsewhere. Considering the paramount need to assess the presence and abundance of vector mosquitoes considering the tropical climate and unique conditions of Miami-Dade County, Florida we hypothesize that the effectiveness and reliability of using BG-Sentinel traps for the surveillance of vector mosquitoes strongly depend on the bait used to attract mosquitoes. Taking that into account, the objective of this study was to compare the effectiveness of BG-Sentinel traps baited with CO_2_ and BG-Lure in Miami-Dade County, Florida.

## Methods

### Study design

**Fig 1:**
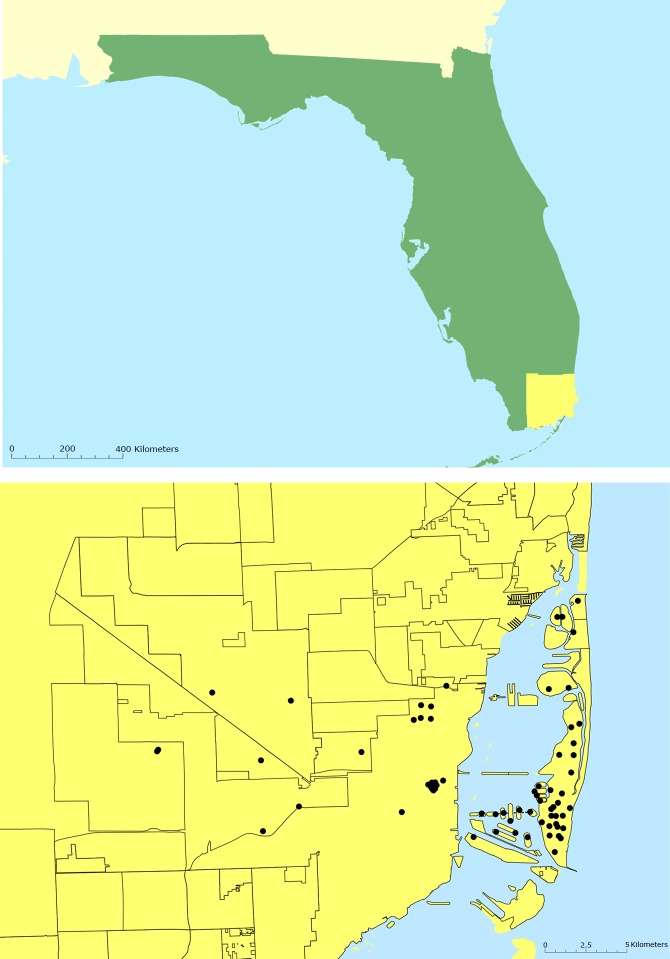
Maps displaying the BG-Sentinel trap locations in Miami-Dade County, Florida. Map above: in green, state of Florida and in yellow Miami-Dade County; Map below: Miami-Dade County, Florida (latitude, 25.761681; longitude, -80.191788).

Collection of mosquitoes was conducted using 72 BG-Sentinel traps (Biogents AG, Regensburg, Germany) across 21 neighborhoods of Miami-Dade County, Florida. The location of the traps was chosen based on areas previously affected by the ZIKV outbreak in Miami, namely Miami Beach, Wynwood and Little River [40]. These areas are still considered to be at higher risk for the introduction of arboviruses due to the increased number of travelers and outdoor activities [41], with much of the surveillance and control efforts directed to control mosquito populations in those areas (Fig 1).

The sampling effort was standardized for all collections. Each BG-Sentinel trap was deployed 4 times from September to October 2018, for 48 hours (24 hours using each bait) once a week for 4 weeks. For the initial 24 hours period, the traps were baited with CO_2_ using containers filled with 1 Kg of dry ice pellets, and then subsequently serviced and immediately baited with BG-Lure (BioGents, Regensburg, Germany) for additional 24 hours. Collected mosquitoes were transported to the Miami-Dade County Mosquito Control Laboratory and subsequently morphologically identified using taxonomic keys [42]. Considering that BG-Sentinel traps mimic a host and, therefore, only actively attract female mosquitoes seeking for blood feeding, the male mosquitoes collected were considered accidental catches and were not included in the analyzes.

Since this study poses less than minimal risk to participants and did not involve endangered or protected species the Institutional Review Board at the University of Miami determined that the study was exempt from institutional review board assessment (IRB Protocol Number: 20161212).

### Data analysis

We performed a Generalized Estimating Equation (GEE) for repeated measures analysis and using Poisson for the dependent variable distribution for mosquito counts. We had traps as the unit, species, and attractor type, and weeks in the longitudinal model. We tested the interaction species by attractor type and it was non-significant, so it was removed from the model. We kept the scale parameter at 1 and did not estimate it. The Quasi-likelihood under Independence Model Criterion (QICC) for the goodness of fit of the model was 5676. Having species in the model allowed us to analyze all the data in one model. The Poisson link was transformed considering Exp(Beta), resulting in the rate ratio. We also used the ANOVA Type III sum of squares method to analyze the number of mosquitoes collected by BG-Sentinel traps baited with different attractants.

The main goal of the Miami-Dade Mosquito control surveillance system is to monitor the relative abundance of primary vectors (i.e., *Ae*. *aegypti* and *Cx*. *quinquefasciatus*) and guide control interventions when needed. However, many other neglected vectors are present in Miami and an effective surveillance system has obligatorily to be able to detect species such as *Cx*. *coronator*, *Cx*. *nigripalpus*, and *Cx*. *erraticus*. Failing in attracting a wide range of species will result in the unfeasibility of using a given mosquito attractant for surveillance purposes in Miami. Therefore, biodiversity patterns and differences in the mosquito assembly for the collections comprising female mosquitoes collected by BG-Sentinel traps using CO_2_ and BG-Lure were analyzed by the Shannon (H) and Simpson (1-D) biodiversity indices [43]. Subsequently, the individual rarefaction curves were generated to estimate sampling sufficiency and the expected occurrence of species for smaller samples. Plots of cumulative species abundance (ln S), Shannon index (H) and log evenness (ln E) (SHE) profiles were also calculated for all collected mosquitoes; changes in the direction of the lines indicate ecological heterogeneity of mosquito assembly [44]. Analyses were carried out with 10,000 randomizations without replacement and a 95% confidence interval using Past software (v.3.16) [45,46]. Fig 1 was produced using ArcGIS 10.2 (Esri, Redlands, CA). Climate data was obtained at the National weather services (available at: https://www.weather.gov/mfl/) (S1 Table).

## Results

**Fig 2:**
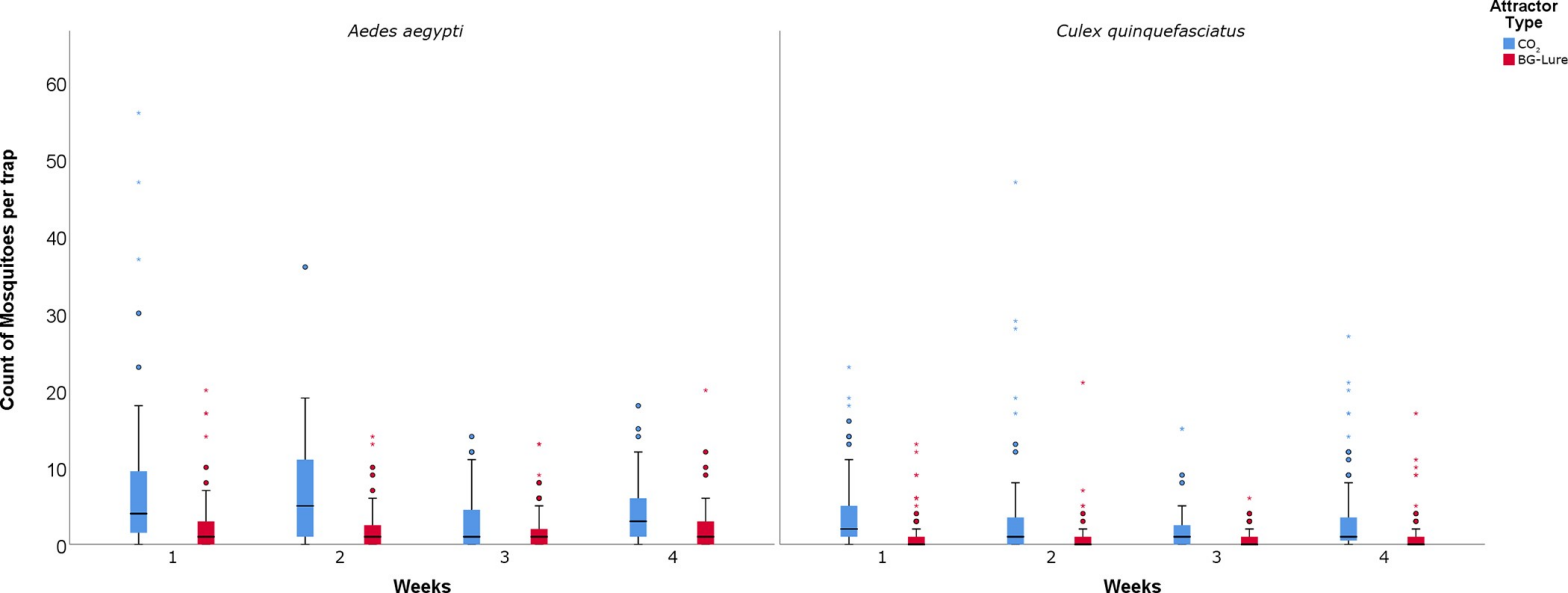
Box-plot graphs of collected *Aedes aegypti* and *Culex quinquefasciatus* over the 4 weeks of collection in Miami-Dade, Florida using BG-Sentinel traps baited with CO_2_ and BG-Lure.

A total of 5,154 female mosquitoes were collected, from which 3,514 were collected by BG-Sentinel traps baited with CO_2_ and 1,640 by BG-Sentinel traps baited with BG-Lure. BG-Sentinel traps baited with CO_2_ collected 12 species of mosquitoes distributed among 4 genera. On the other hand, BG-Sentinel traps baited with BG-Lure collected 5 species of mosquitoes from 3 genera. *Aedes aegypti* was the most collected species totaling 2,674 specimens collected, from which 1,730 were collected by traps baited with CO_2_ and 944 by traps baited with BG-Lure. *Culex quinquefasciatus* was the second most abundant mosquito species, adding a total of 2,213 specimens collected, from which 1,529 were collected by traps baited with CO_2_ and 684 by traps baited with BG-Lure (Table 1, Fig 2).

**Table 1:** Mosquitoes collected by the 72 BG-Sentinel traps baited with CO_2_ and BG-Lure in Miami-Dade County, Florida.

	CO_2_					BG-Lure				
	Week 1	Week 2	Week 3	Week 4	Total CO_2_	Week 1	Week 2	Week 3	Week 4	Total BG-Lure
*Aedes aegypti*	543	478	292	417	1730	276	197	237	234	944
*Aedes albopictus*	6	3	1		10	2	2		5	9
*Aedes taeniorhynchus*		1			1					
*Aedes tortilis*	41	2			43					
*Aedes triseriatus*	3	3	1	1	8					
*Anopheles quadrimaculatus*	1				1					
*Culex coronator*	16	9	7	5	37					
*Culex erraticus*	1				1					
*Culex nigripalpus*	5	59	7		71					
*Culex quinquefasciatus*	422	417	240	450	1529	234	150	171	129	684
*Wyeomyia mitchelli*	25	34	2		61		1			1
*Wyeomyia vanduzeei*	5	10	7		22		2			2
Total	1068	1016	557	873	3514	512	352	408	368	1640

**Fig 3:**
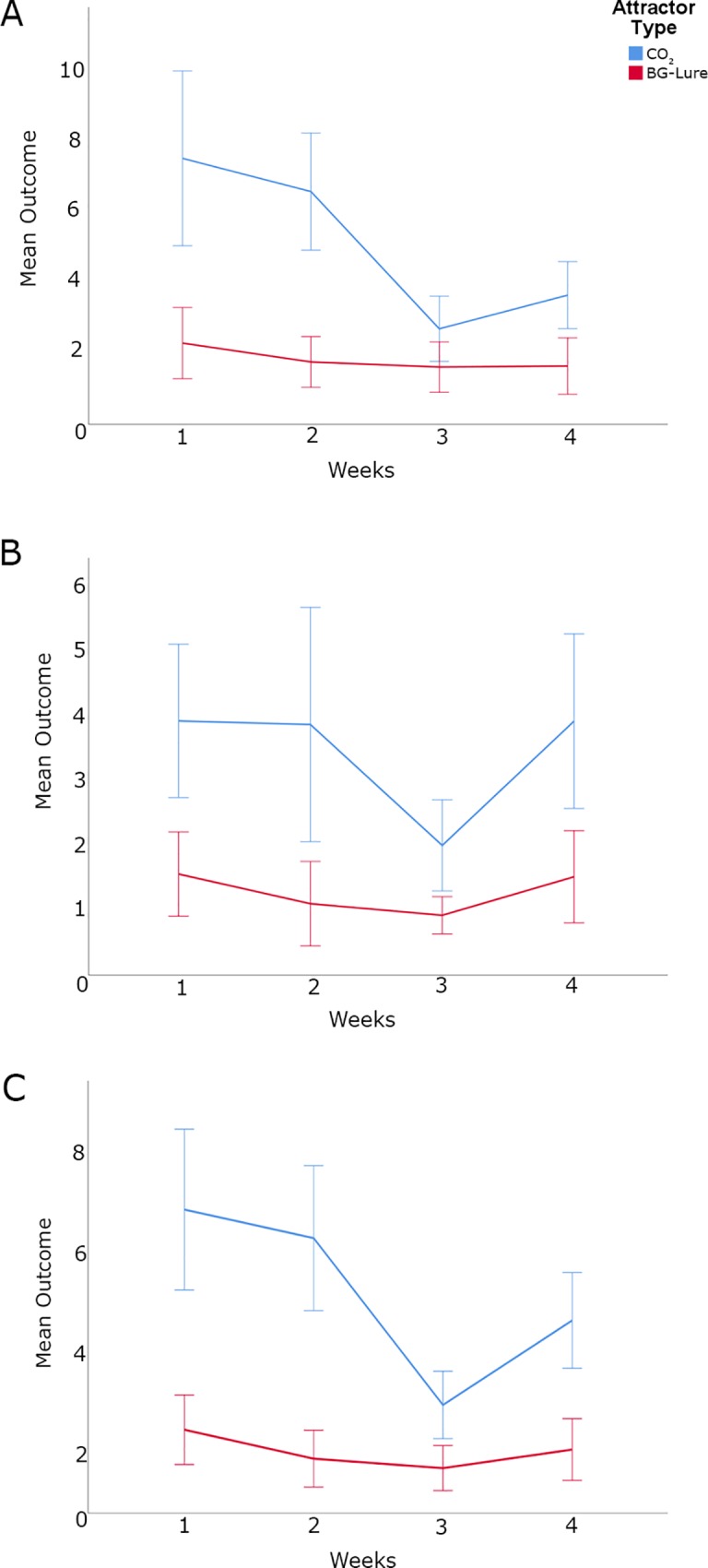
Graphs of means of collected mosquitoes over the 4 weeks of collections in Miami-Dade, Florida using BG-Sentinel traps baited with CO_2_ and BG-Lure. (A) *Aedes aegypti*; (B) *Culex quinquefasciatus*; (C) sum of *Aedes aegypti* and *Culex quinquefasciatus*.

Results from the Generalized Estimating Equation models indicated that more than twice as many *Ae*. *aegypti* and *Cx*. *quinquefasciatus* were attracted to traps baited with CO_2_ than to traps baited with BG-Lure (*P* = 0.001; exp(0.984) = 2.67). Furthermore, despite of the *Cx*. *quinquefasciatus* relative abundance being consistently higher than for *Ae*. *aegypti* according to the Miami-Dade Mosquito Control surveillance database (unpublished results) the comparison of the attractiveness of CO_2_ and BG-Lure to *Ae*. *aegypti* and *Cx*. *quinquefasciatus* was found to be non-significant. *Ae*. *aegypti* was significantly more attracted to CO_2_ and BG-Lure, yielding almost 70% more specimens collected in the traps (*P* = 0.000; exp(0.512) = 1.67). The difference between CO_2_ and BG-Lure was also significantly different adjusting for species. After the removal of the effects of the difference of species in the data variability, were there still differences between attractants (Table 2, S2 and S3 Tables, Fig 3).

**Table 2:** Results of Generalized Estimating Equation models for *Aedes aegypti* and *Culex quinquefasciatus* collected by BG-Sentinel traps baited with CO_2_ and BG-Lure.

Coefficients	Adjusted Rate Ratio	95%LCL	95%UCL
Week 1 vs. 4*	1.41	1.12	1.76
Week 2 vs. 4	1.23	1	1.51
Week 3 vs. 4*	0.68	0.55	0.85
CO_2_ vs BG-Lure*	2.67	2.04	3.51
*Aedes aegypti* vs. Culex quinquefasciatus*	1.67	1.27	2.18

* = significant values at an alpha level of 0.05.

Adjusted Rate Ration are significant different than 1. Dependent variable: outcome; Model: (Intercept), weeks, bait type, species; a. set to zero because this parameter is redundant; scale = 1; LCL = lower confidence interval. UCL = upper confidence interval.

**Fig 4:**
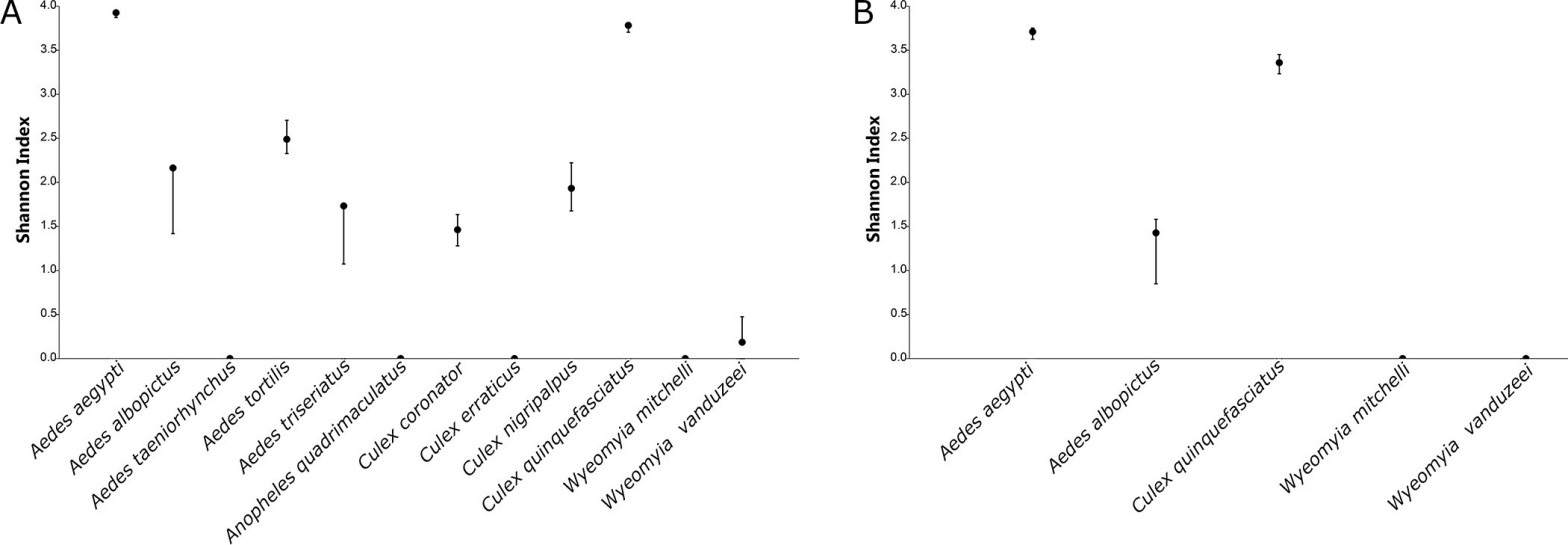
Shannon (H) index for mosquitoes collected in Miami-Dade County, Florida using BG-Sentinel traps baited with (A) CO_2_ and (B) BG-Lure.

Despite the difference in the species richness and abundance comprising the mosquitoes collected by BG-Sentinel traps baited with CO_2_ and BG-Lure, the biodiversity indices indicated a similar scenario, in which *Ae*. *aegypti* and *Cx*. *quinquefasciatus* were the most dominant species. The average of the Shannon (H) index of BG-Sentinel traps baited with CO_2_ was 1.472 (95% CI: 1.295–1.557) and of BG-Sentinel traps baited with BG-Lure was 1.699 (95% CI: 1.508–1.757). *Aedes aegypti* collected by BG-Sentinel traps baited with CO_2_ yielded the highest value in the Shannon (H) index, 3.926 (95% CI: 3.872–3.942), followed by *Cx*. *quinquefasciatus* 3.783 (95% CI: 3.705–3.815). Similar results were obtained by BG-Sentinel traps baited with BG-Lure, in which *Ae*. *aegypti* yielded the highest values 3.711 (95% CI: 3.624–3.751), followed by *Cx*. *quinquefasciatus* 3.360 (95% CI: 3.235–3.456) (Fig 4).

**Fig 5:**
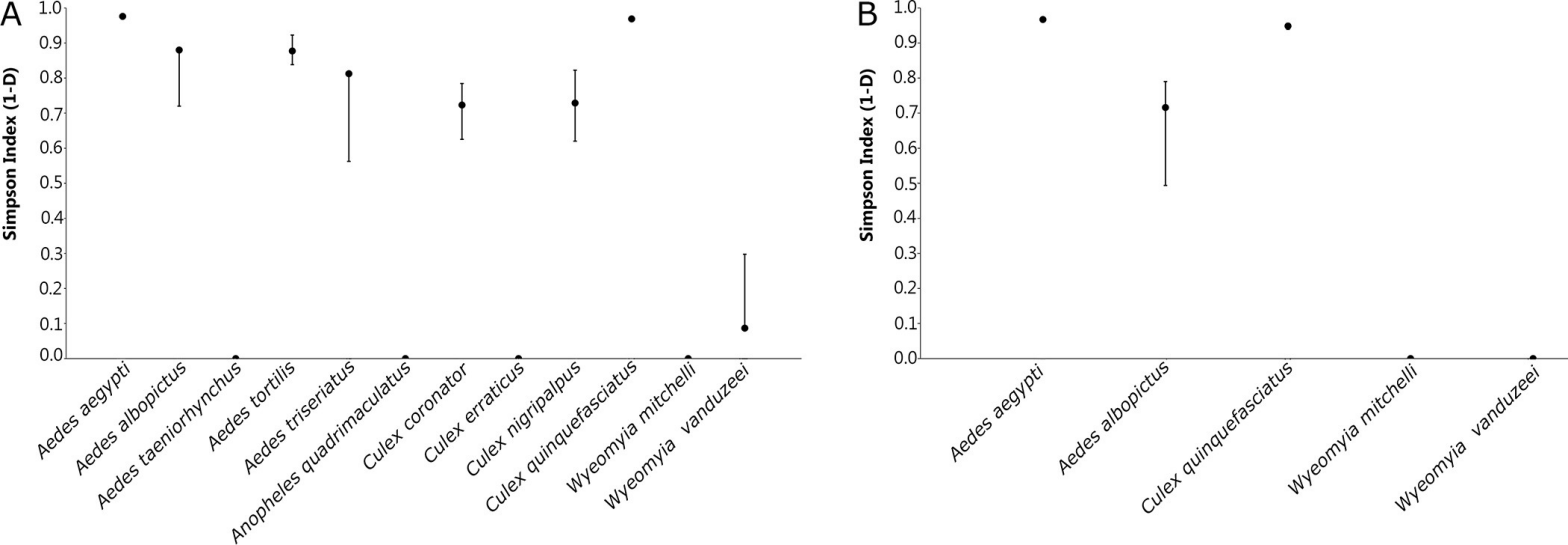
Simpson (1-D) index for mosquitoes collected in Miami-Dade County, Florida using BG-Sentinel traps baited with (A) CO_2_ and (B) BG-Lure.

*Aedes aegypti* yielded the highest value in the Simpson (1-D) index, 0.976 (95% CI: 0.974–0.976) in BG-Sentinel traps baited with CO_2_, followed by *Cx*. *quinquefasciatus* 0.968 (95% CI: 0.964–0.970). BG-Sentinel traps baited with BG-Lure had similar results, *Ae*. *aegypti* was the most dominant species according to the Simpson (1-D) index, 0.967 (95% CI: 0.961–0.969), followed by *Cx*. *quinquefasciatus* 0.948 (95% CI: 0.939–0.956) (Fig 5).

**Fig 6:**
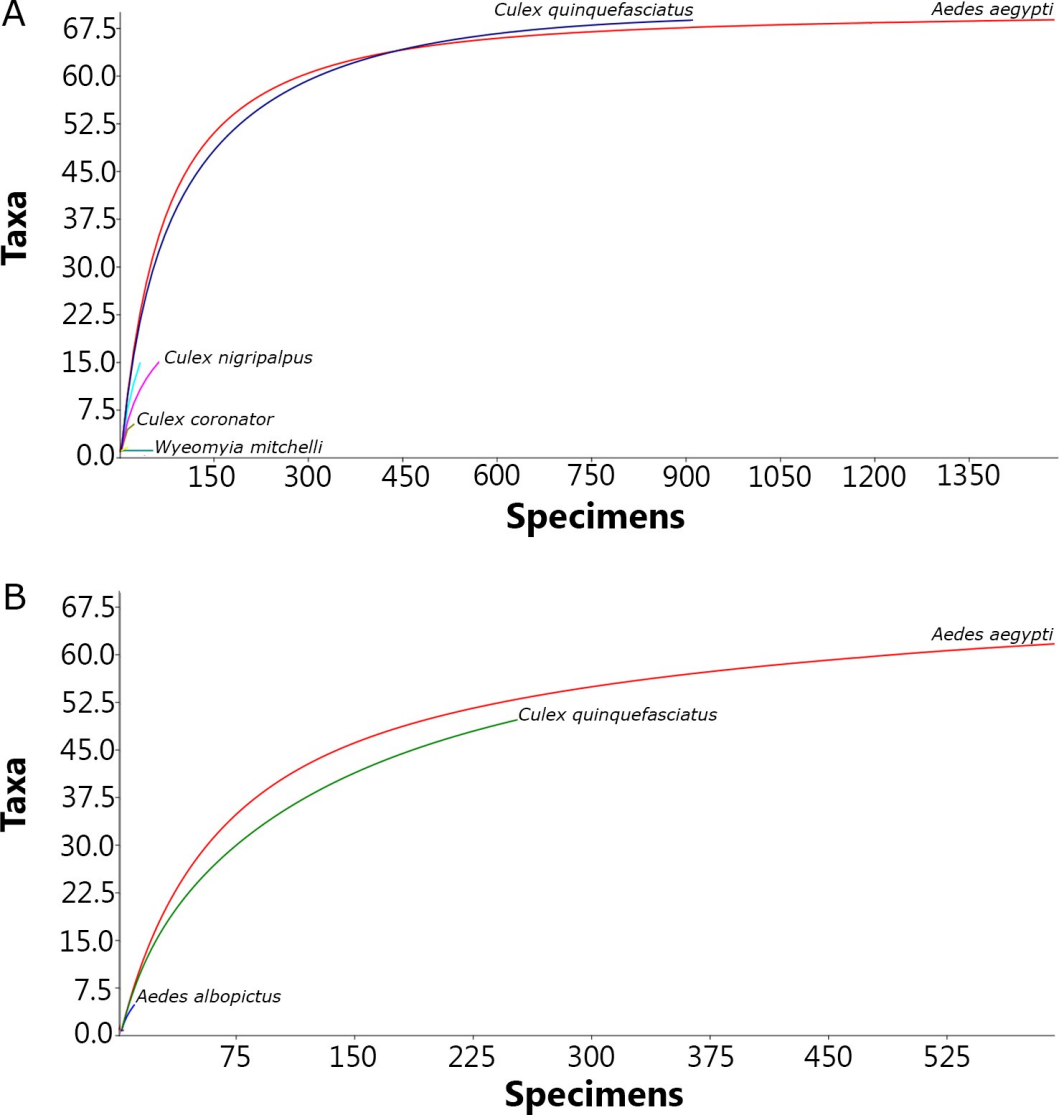
Individual rarefaction curves of mosquitoes collected in Miami-Dade, Florida using BG-Sentinel traps baited with (A) CO_2_ and (B) BG-Lure.

The individual rarefaction curves resulted in two distinct scenarios. In the same timeframe and with identical sampling effort, BG-Sentinel traps baited with CO_2_ yielded highly asymptotic curves for *Ae*. *aegypti* and *Cx*. *quinquefasciatus* indicating a high degree of confidence for assessing the relative abundance of these species. The individual rarefaction curves for BG-Sentinel traps baited with BG-Lure, on the other hand, did not reach the asymptote for *Cx*. *quinquefasciatus* and reached a moderate asymptote curve for *Ae*. *aegypti*. These results are indicating that sampling sufficiency was not reached by traps using BG-Lure as bait (Fig 6).

**Fig 7:**
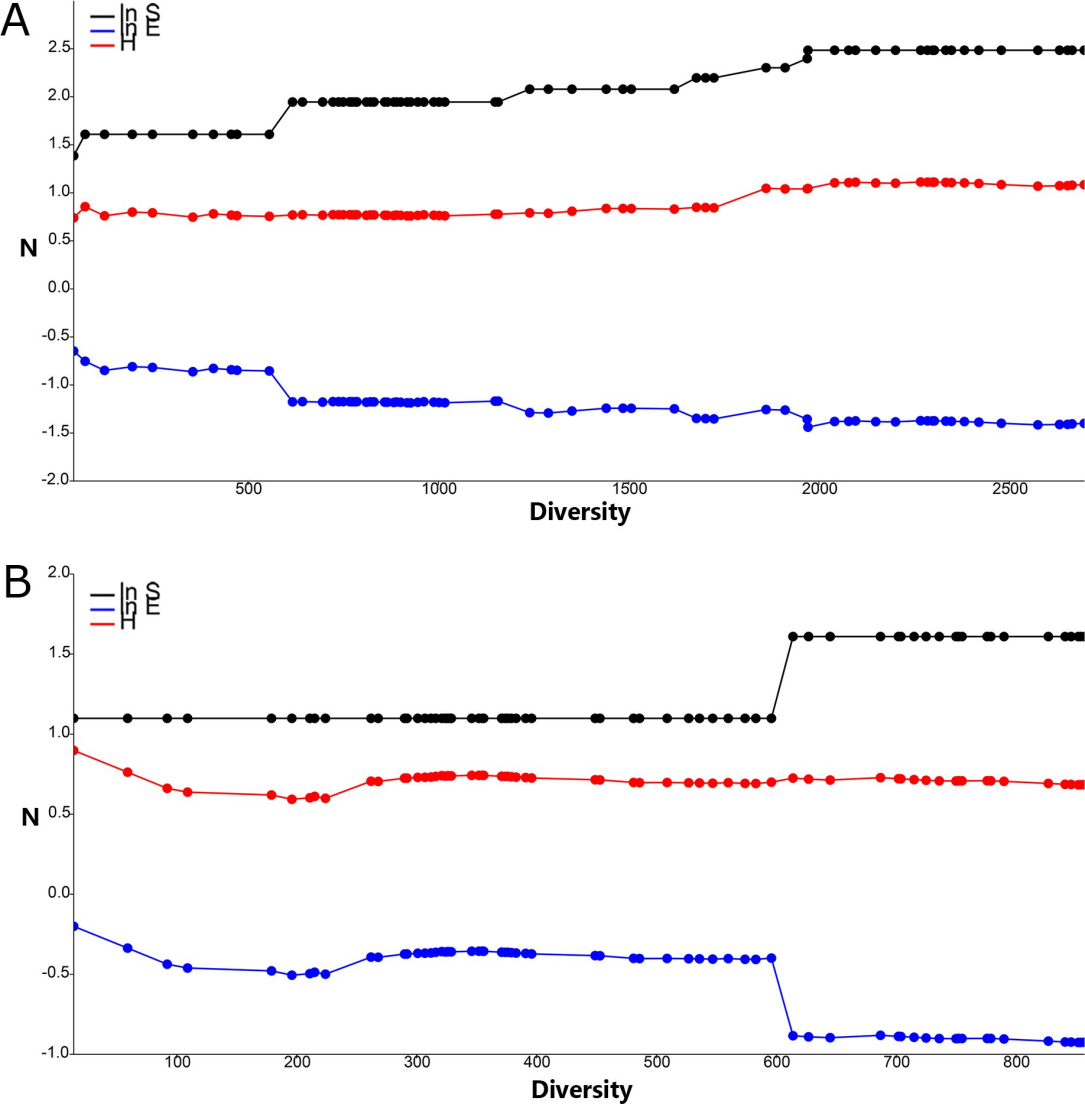
Plots of cumulative species abundance (ln S), Shannon index (H) and log evenness (ln E) profiles (SHE) of mosquitoes collected in Miami-Dade, Florida using BG-Sentinel traps baited with (A) CO_2_ and (B) BG-Lure.

The changes in the direction of the lines in the cumulative SHE analysis exposed two different scenarios comprising species composition, diversity and evenness. BG-Sentinel traps baited with CO_2_ displayed substantial deviations from a straight line in the SHE analysis indicating a higher degree of heterogeneity in the composition of species. It was possible to observe multiple increases in value in the cumulative species abundance (Ln S) of traps baited with CO_2_, indicating an increased number of species collected when compared to traps baited with BG-Lure. A similar result was found for the log evenness (ln E), in which traps baited with BG-Lure displayed an unbalanced assembly of mosquitoes with mostly *Ae*. *aegypti* and *Cx*. *quinquefasciatus* comprising all collected specimens. BG-Sentinel traps baited with CO_2_ had the opposite results considering the Shannon index (H) with its gradual increase, contrasting with the decrease for traps baited with BG-Lure (Fig 7).

## Discussion

Mosquito control programs rely on surveillance programs to guide their control operations. The relative abundance of adult mosquitoes in a given area is widely used to trigger control efforts such as management of breeding sites and spraying of insecticides. Therefore, achieving sampling sufficiency to consistently, correctly and reliably assess the relative abundance of adult mosquitoes is critical to prevent VBD outbreaks and protect residents and tourists. Our results indicated that BG-Sentinel traps baited with BG-Lure underestimated the richness and abundance of species when compared to the results obtained by BG-Sentinel traps baited with CO_2_. Furthermore, traps baited with CO_2_ collected twice as many specimens and species providing a more realistic panorama of the mosquito assembly and relative abundance of adult mosquitoes.

Even though the results are indicating that BG-Sentinel traps baited with BG-Lure consistently collected *Ae*. *aegypti*, even though in fewer numbers when compared to traps baited with CO_2_, the same was not true for the remaining species. Traps baited with BG-Lure were substantially less effective in collecting mosquitoes with low abundances such as *Wyeomyia mitchelli* and *Wyeomyia vanduzeei* that were only collected once (week 2), contrasting with traps baited with CO_2_, in which they were collected in three occasions (weeks 1, 2 and 3). Similar results were found for *Cx*. *coronator* and *Cx*. *nigripalpus*, in which neither species was collected by traps baited with BG-Lure but were consistently collected by traps baited with CO_2_.

Failing to collect important vector species can lead to the incorrect underestimation of their relative abundance and erroneously lead to inaccurately guided mosquito control operations or, in this case, the lack of it. *Culex* species, such as *Cx*. *coronator* and *Cx*. *nigripalpus* are primary vectors of WNV and failing to detect them may expose the human population to vector-borne pathogens [24,27,47]. Migratory birds, the natural reservoir of WNV, often stop in suburban and urban areas when they may come in contact with *Culex* mosquitoes, with the potential of triggering an outbreak [48–50].

Furthermore, an effective mosquito surveillance system based on relative abundance of adult mosquitoes has to account not only for *Ae*. *aegypti*, but if possible to all vector species in the region. Many pathogens are circulating under the radar that can be vectored by species from the genera *Culex* and *Wyeomyia*, among many others [51,52]. In a more pessimistic scenario, one can also speculate that there are many more arboviruses than we are aware of, such as ZIKV was once in the past [53].

We were not able to collect data across all weather and season variations that would provide more data on the effectiveness of BG-Sentinel traps baited with CO_2_ and BG-Lure in assessing mosquito abundance and species richness.

## Conclusion

According to our results, BG-Sentinel traps baited with CO_2_ were more effective in assessing the relative abundance of adult vector mosquitoes in comparison with BG-Sentinel traps baited with BG-Lure. Traps baited with CO_2_ provided more trustworthy results, collecting not only more specimens, but also more species in a more consistent pattern. For this reason, we believe that BG-Sentinel traps baited only with BG-Lure should not be used for the surveillance of vector mosquitoes other than *Ae*. *aegypti*. In the specific case of Miami, in which many vector mosquitoes are present, and the surveillance program is not limited to only survey *Ae*. *aegypti*, the surveillance system has to be able to detect and provide reliable estimates of the presence and abundance of several species of vector mosquitoes rendering the use of BG-Sentinel traps baited with BG-Lure significantly less effective and, therefore, not recommended.

## Supporting information

S1 Fig Map of Miami-Dade County, Florida displaying the BG-Sentinel trap locations in relation to neighborhoods. (TIF)

S1 Table Climate variation in Miami-Dade County from September to October 2018. (DOCX)

S2 Table Analysis of variance for mosquitoes collected by BG-Sentinel traps baited with CO2 and BG-Lure. (DOCX)

S3 Table Estimated marginal means and standard errors from the Analysis of variance for mosquitoes collected by BG-Sentinel traps baited with CO_2_ and BG-Lure. (DOCX)
