# Supplementary figures and images for: Assessment of the effectiveness of BG-Sentinel traps baited with CO2 and BG-Lure for the surveillance of vector mosquitoes in Miami-Dade County, Florida
Source: PLoS One. 2019 Feb 22;14(2):e0212688. doi: 10.1371/journal.pone.0212688 (PMC6386269; doi:10.1371/journal.pone.0212688)

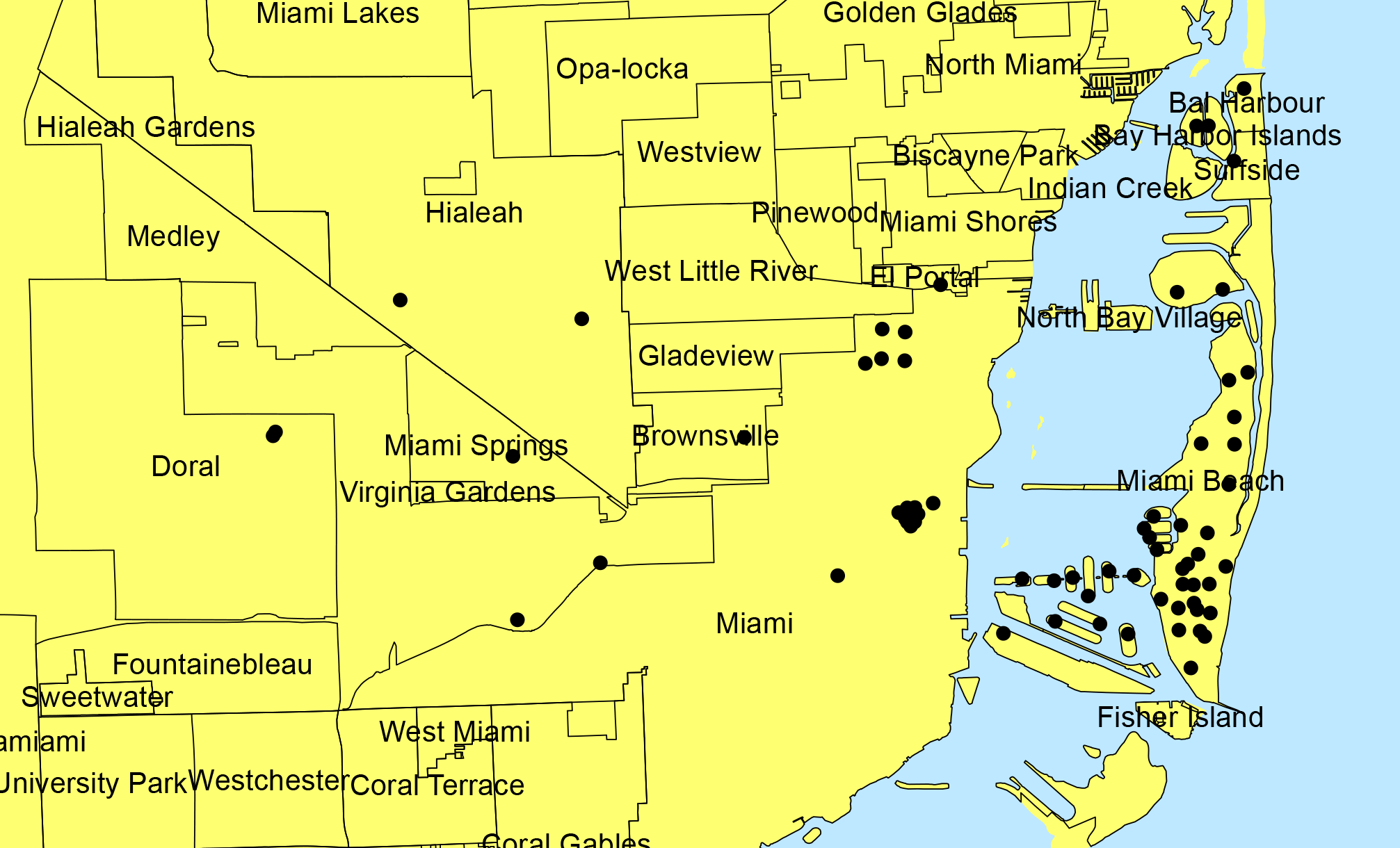

Supplement: S1 Fig — (TIF) [file pone.0212688.s001.tif]
