# Supplementary material for: Assessment of the effectiveness of BG-Sentinel traps baited with CO2 and BG-Lure for the surveillance of vector mosquitoes in Miami-Dade County, Florida
Source: PLoS One. 2019 Feb 22;14(2):e0212688. doi: 10.1371/journal.pone.0212688 (PMC6386269; doi:10.1371/journal.pone.0212688)
Supplement: S1 Table — (DOCX) [file pone.0212688.s002.docx]

**S1 Table.** Climate variation in Miami-Dade County from September to October 2018.

|  | 11-Sep | 12-Sep | 18-Sep | 19-Sep | 25-Sep | 26-Sep | 2-Oct | 3-Oct |
| --- | --- | --- | --- | --- | --- | --- | --- | --- |
| Temp Max (^o^C) | 32 | 33 | 32 | 32 | 32 | 32 | 31 | 31 |
| Temp Min (^o^C) | 26 | 27 | 26 | 26 | 26 | 27 | 24 | 26 |
| Wind Max (K/h) | 8 | 7 | 11 | 9 | 11 | 12 | 16 | 12 |
| Wind Min (K/h) | 2 | 1 | 4 | 2 | 2 | 6 | 6 | 6 |
