# Supplementary material for: Assessment of the effectiveness of BG-Sentinel traps baited with CO2 and BG-Lure for the surveillance of vector mosquitoes in Miami-Dade County, Florida
Source: PLoS One. 2019 Feb 22;14(2):e0212688. doi: 10.1371/journal.pone.0212688 (PMC6386269; doi:10.1371/journal.pone.0212688)
Supplement: S2 Table — (DOCX) [file pone.0212688.s003.docx]

**S2 Table.** Analysis of variance for mosquitoes collected by BG-Sentinel traps baited with CO_2_ and BG-Lure.

| **Variable** | **Type III Sum of Squares** | **Mean Square** | **F** | ***P*** |
| --- | --- | --- | --- | --- |
| Time | 515.79 | 515.79 | 17.636 | <0.001 |
| Time by attractant | 293.686 | 293.686 | 10.042 | 0.002 |
