# Supplementary material for: Assessment of the effectiveness of BG-Sentinel traps baited with CO2 and BG-Lure for the surveillance of vector mosquitoes in Miami-Dade County, Florida
Source: PLoS One. 2019 Feb 22;14(2):e0212688. doi: 10.1371/journal.pone.0212688 (PMC6386269; doi:10.1371/journal.pone.0212688)
Supplement: S3 Table — (DOCX) [file pone.0212688.s004.docx]

**S3 Table.** Estimated marginal means and standard errors from the Analysis of variance for mosquitoes collected by BG-Sentinel traps baited with CO_2_ and BG-Lure.

| **Week** | **Lure** | **Mean** | **Std. Error** | **95% Confidence Interval** | |
| --- | --- | --- | --- | --- | --- |
|  |  |  |  | **Lower Bound** | **Upper Bound** |
| 1 | CO_2_ | 7.542 | 0.914 | 5.734 | 9.349 |
|  | BG-Lure | 2.479 | 0.921 | 0.659 | 4.299 |
| 2 | CO_2_ | 6.639 | 0.617 | 5.419 | 7.859 |
|  | BG-Lure | 2 | 0.621 | 0.772 | 3.228 |
| 3 | CO_2_ | 2.903 | 0.395 | 2.123 | 3.683 |
|  | BG-Lure | 1.775 | 0.397 | 0.989 | 2.56 |
| 4 | CO_2_ | 3.819 | 0.424 | 2.982 | 4.657 |
|  | BG-Lure | 1.859 | 0.427 | 1.015 | 2.703 |
